# Draft Genome Sequence of Lactarius deliciosus Strain CBS 582.63 from the CBS-KNAW Culture Collection

**DOI:** 10.1128/MRA.01485-19

**Published:** 2020-05-21

**Authors:** Jia Ean Goh, Ahmad Yamin Rahman, Ranjeev Hari, Mei Peng Lim, Nazalan Najimudin, Wai Sum Yap, Yong Hui Tan, Baskaran Gunasekaran, Hok Chai Yam

**Affiliations:** a Faculty of Applied Sciences, UCSI University, Kuala Lumpur, Malaysia; b School of Computer Sciences, Universiti Sains Malaysia, Pulau Pinang, Malaysia; c School of Data Sciences, Perdana University, Serdang, Malaysia; d Robarts Research Institute, University of Western Ontario, London, Canada; University of Maryland School of Medicine

## Abstract

A type strain of Lactarius deliciosus was obtained from the CBS-KNAW culture collection. The mycelium was cultured using potato dextrose agar, and the extracted genomic DNA was subjected to PacBio genome sequencing. Upon assembly and annotation, the genome size was estimated to be 54 Mbp, with 12,753 genes.

## ANNOUNCEMENT

Edible *Lactarius* mushrooms, also known as milk cap mushrooms, are commonly found in the Northern Hemisphere. They produce natural rubber (high-molecular-weight *cis*-polyisoprene), as do some plants (1, 2). Lactarius deliciosus, commonly known as the saffron milk cap mushroom, belongs to the family *Russulaceae*, of the *Basidiomycota* subdivision of *Fungi*.

Mushroom strain CBS 582.63 was purchased from the CBS-KNAW culture collection (Netherlands). *L. deliciosus* was cultivated in potato dextrose agar (PDA) medium as described by Hall and Wang (3). The mycelium was cultured at room temperature (27°C) under dark conditions until enough mass was obtained.

Genomic DNA (gDNA) was isolated by using Genomic-tips with protocols recommended by the manufacturer (Qiagen, Germany). The isolated gDNA sample was subjected to high-throughput PacBio Sequel sequencing. The sequencing used the v2 chemistry provided by PacBio utilizing 4 single-molecule real-time (SMRT) cells. A 20-kb library with a 5-kb cutoff was prepared using the protocols of the SMRTbell template prep kit v1.0 according to the manufacturer’s recommendations (PacBio, Menlo Park, CA). Approximately 3 Gb of sequence data was generated from the sequencing, and a sequence coverage of about 61× was obtained. The SMRT sequencing yielded a mean read length of 7,965 bp and 386,212 total reads.

The raw sequences were assembled by using fast mapping, error correction, and the *de novo* assembly tool MECAT v1.2 (4), followed by quality assessment by QUAST v4.6 (5). Default parameters were used for all software unless otherwise stated. Genome assembly produced a total length of 54 Mbp with 841 contigs, an *N*_50_ value of 116,430 bp, and a GC content of approximately 51.98%. Fast alignment-free computation of whole-genome average nucleotide identity was performed using FastANI v1.3 (6), and the results indicated that the assembled genome sequence showed the highest identity with the genome sequences of *Lactarius deliciosus* strains deposited in the Joint Genome Institute (JGI).

Annotation of the assembled genomic sequences of *L. deliciosus* was performed with MAKER2 v2.31 (7). This tool runs independent gene callers and creates consensus predictions based on collective evidence. Three separate gene predictors, AUGUSTUS v2.5.5 (8), SNAP v2013-11-29 (9), and GeneMark-ES v2.3 (10), were also used to predict evidence. *L. deliciosus* expressed sequence tag (EST) data from the Joint Genome Institute (JGI) were used as transcriptomic data, while the protein sequence was used as annotation reference data (11). The annotation resulted in 12,753 genes.

### Data availability.

The genome of *Lactarius deliciosus* CBS 582.63 has been deposited in DDBJ/ENA/GenBank under the accession number VHKJ00000000. This version of the project can be found under accession number VHKJ01000000. The raw data have been deposited in the SRA under accession number PRJNA550006.
